# Late presentation of paediatric patients to clinics: a major barrier to uptake of laboratory services in nigeria

**DOI:** 10.1186/2047-2994-4-S1-P265

**Published:** 2015-06-16

**Authors:** CO Okolo

**Affiliations:** 1 HIV/AIDS, John Snow Incorporated, Nigeria

## Introduction

In Nigeria, paediatric patients present to health clinics at critical states that leave the medical doctors with no other choice but to blindly prescribe drugs without proper diagnostic tests.

## Objectives

This study was aimed at determining effect of late-presentation of paediatric malaria cases to the hospital on uptake of laboratory services.

## Methods

Conducted study at the Medical Centre, University of Nigeria, Enugu Campus. Reviewed medical records contents of fifty-six paediatric patients from January 22 to March 23, 2014. Records were taken during clinic days at the OPD section. Malaria parasite test showing 3+++ and PCV below 39% of the patients involved were used as a major basis for sampling. The following inclusion criteria were used: age bracket of 1-12years, late presentation, very weak with severe fever, malaria test of 3+++ and PCV below 39% while exclusion criteria included adults, early presenters and patients presenting symptoms not related to malaria. The following were the main instruments used; structured questionnaires, medical records and Micro-haematocrit centrifuge.

## Results

55% the patients admitted did not use laboratory services until their first line of anti-malaria therapy ended; their PCV reading was recorded as 39%. 45% of the patients presented to the medical centre during the weekend when the Lab Scientist was not on duty; they recorded lower PCV reading of 38% during the week day when the Lab scientist had resumed.

## Conclusion

Late presentation which comes either in the form of:

- Patients presenting late to the clinic with severe malaria symptoms. This leads to anti-malaria therapy for the paediatric patients without the necessary diagnostic tests in a bid to save their lives, or

- Patients presenting to the medical centre during the weekend when the only Lab Scientist working at the medical centre is off-duty.

All these act as barriers to uptake of laboratory services.

## Disclosure of interest

None declared.

